# Invasive mechanical ventilation, functional decline at pediatric intensive care unit discharge, and post-discharge mortality in critically ill pediatric cancer patients: a retrospective cohort study

**DOI:** 10.62675/2965-2774.20260121

**Published:** 2026-09-17

**Authors:** Beatriz Silva Menezes da Cunha-Martins, Rachel Silva Menezes da Cunha, Vanessa Soares Lanziotti, Antonio Paulo Nassar

**Affiliations:** 1 Instituto Nacional de Câncer Hospital do Câncer I Technical-Assistance Division Rio de Janeiro RJ Brazil Rehabilitation Session, Technical-Assistance Division, Pediatric Oncology Service, Hospital do Câncer I, Instituto Nacional de Câncer - Rio de Janeiro (RJ), Brazil.; 2 Universidade Federal do Rio de Janeiro Pediatric Intensive Care Unit Instituto de Puericultura e Pediatria Martagão Gesteira Rio de Janeiro RJ Brazil Pediatric Intensive Care Unit, Instituto de Puericultura e Pediatria Martagão Gesteira, Universidade Federal do Rio de Janeiro - Rio de Janeiro (RJ), Brazil.; 3 Intensive Care Unit A.C. Camargo Cancer Center São Paulo SP Brazil Intensive Care Unit, A.C. Camargo Cancer Center - São Paulo (SP), Brazil.

**Keywords:** Respiration, artificial, Neoplasms, Functional status, Morbidity, Mortality, Intensive care units, pediatric

## Abstract

**Objective:**

To evaluate the association between invasive mechanical ventilation use, functional capacity, and 12-month mortality in critically ill children with cancer after a pediatric intensive care unit stay.

**Methods:**

We conducted a single-center, retrospective cohort study of critically ill children with cancer admitted to the pediatric intensive care unit from January 2018 to December 2022. We applied the Functional Status Scale to classify functional capacity before pediatric intensive care unit admission and at discharge, and to determine the onset of new morbidities. We assessed the association between invasive mechanical ventilation use and the development of new morbidity (defined as an increase of at least three points in the Functional Status Scale at discharge, and as an increase of at least two points in any domain of Functional Status Scale at discharge), between invasive mechanical ventilation use and 12-month mortality after pediatric intensive care unit discharge, and of the onset of new morbidities and mortality during the same period.

**Results:**

We included 423 patients, of whom 176 (41%) used invasive mechanical ventilation. The frequency of new morbidities was higher in the mechanically ventilated patients (39.2% *versus* 4.8%; p < 0.001). Invasive mechanical ventilation use was associated with the development of new morbidity (adjusted OR = 13; 95%CI = 6.6 - 27.3; p < 0.001). New morbidity was associated with increased mortality at 12 months after pediatric intensive care unit discharge (adjusted OR = 2.1; 95%CI = 1.2 - 3.7; p = 0.013), but mechanical ventilation was not.

**Conclusion:**

Critically ill children with cancer who required invasive mechanical ventilation during pediatric intensive care unit stay had 13-fold higher odds of developing new morbidities. The onset of new morbidity was associated with doubled odds of death within 12 months after pediatric intensive care unit discharge. However, invasive mechanical ventilation use was not directly associated with 12-month mortality.

## INTRODUCTION

Over the past 30 years, advances in clinical and surgical treatments, along with enhanced supportive care, have increased survival rates for children with cancer.^(1)^ However, these patients may develop several complications related to the disease and/or oncologic treatment, including severe conditions that require intensive care support. Approximately 40% of children with cancer experience at least one admission to the pediatric intensive care unit (ICU) during their illness.^(2)^

Critically ill children are at risk of developing or worsening physical, cognitive, emotional, social, and family impairments during or after critical illness — collectively termed pediatric post-intensive care syndrome (PICS-P). Its manifestations vary according to the child's developmental stage and pre-existing complex chronic conditions. Affected domains may include respiratory function, mobility, and muscle strength.^(3,4)^

Critically ill children with cancer are at higher risk of functional disabilities after a pediatric ICU stay.^(5)^ Since invasive mechanical ventilation (IMV) contributes to PICS-P and to short- and long-term mortality in critically ill children,^(2,6,7)^ its impact may be even greater in cancer patients. However, data on these associations in critically ill pediatric cancer patients remain scarce. This study aimed to evaluate the association between IMV use and functional capacity at pediatric ICU discharge, as well as the association of these variables with 12-month mortality among critically ill children with cancer who survived the pediatric ICU stay.

## METHODS

This retrospective, single-center cohort study was conducted in the pediatric ICU of the *Instituto Nacional de Câncer* (INCA) in Rio de Janeiro (RJ), a public tertiary cancer center. The pediatric ICU has six beds and receives approximately 145 admissions per year, and most patients are from Rio de Janeiro state. We included all patients admitted to the pediatric ICU between January 1st, 2018, and December 31, 2022. The institutional Research Ethics Committee approved the study (CAAE: 68197023.9.0000.5274), and it granted a waiver of informed consent. This report followed the Strengthening the Reporting of Observational Studies in Epidemiology (STROBE) guidelines for cohort studies.^(8)^

The study included patients admitted for clinical or surgical conditions, with an age of 18 years or younger, and with a diagnosis of cancer confirmed by pathological or imaging exams. We excluded patients on IMV before pediatric ICU admission, those with a pediatric ICU stay of less than 24 hours, those readmitted to the pediatric ICU during the same hospital stay, and those who died during the pediatric ICU stay.

We extracted the following data from the patients’ electronic medical records: sex, age at admission, type of cancer, presence of metastasis for solid tumors, anticancer treatments before and during pediatric ICU stay, pediatric ICU admission type, source of admission, Pediatric Risk of Mortality (PRISM) I score,^(9)^ use of ventilatory support (via orotracheal tube and tracheostomy), length of IMV use, time from pediatric ICU admission to intubation, physiotherapy team follow-up during pediatric ICU stay, pediatric ICU and hospital length of stay (LOS), and hospital outcome (discharge or death). We also recorded survival status at 12 months after pediatric ICU discharge. We collected data using REDCap forms (Vanderbilt University).^(10)^ We categorized cancer diagnoses according to the International Classification of Childhood Cancer, 3rd edition, update 2017 (ICCC-3-2017).^(11)^ The source of admission was determined by the hospital sector where patients were hospitalized prior to pediatric ICU admission. According to institutional clinical practice, we initiated physiotherapy based on clinical stability criteria and each patient's individual needs, as recommended in the literature.^(12)^

We used the Functional Status Scale (FSS) to classify patients’ functional capacity before pediatric ICU admission and at discharge, based on medical records. The FSS consists of six domains (mental status, sensory functioning, communication, motor functioning, feeding, and respiratory status), each scored from one (normal) to five (very severe dysfunction). The patient's overall functional capacity was categorized as follows: normal (6 - 7 points), mild dysfunction (8 - 9 points), moderate dysfunction (10 - 15 points), severe dysfunction (16 - 21 points), and very severe dysfunction (22 - 30 points).^(13)^

### Exposure and outcomes

Our exposure of interest was any requirement of IMV during pediatric ICU stay. Our primary outcome was the onset of a new morbidity at pediatric ICU discharge. We defined new morbidity as an increase of three or more points in the total FSS score between admission and discharge (Definition 1). We also assessed a secondary definition of new morbidity as an outcome: an increase of two or more points in any domain between the two assessments (Definition 2).^(14)^

### Statistical analysis

We conducted statistical analyses using IBM SPSS Statistics (version 31.0; IBM Corp., Armonk, NY, USA) and RStudio (R Core Team, R Foundation for Statistical Computing).

Categorical variables are shown as absolute counts and relative frequencies and compared using the Chi-squared test. Continuous variables are presented as medians with interquartile ranges (25 - 75% IQR) and compared with the Mann-Whitney U test between mechanically and non-mechanically ventilated patients. Finally, we applied the Wilcoxon signed-rank test to compare total FSS scores at admission and discharge within each group.

We used Kaplan-Meier survival curves and log-rank tests to compare the time from pediatric ICU discharge to death between patients who developed new morbidity and those who did not, using both definitions, as well as between patients who received IMV and those who did not.

We conducted multivariate logistic regression models to examine the association between IMV use and new morbidity at pediatric ICU discharge for both definitions. We assessed the association between IMV and new morbidity in unadjusted and adjusted models for PRISM I score, pediatric ICU admission type, and total FSS score at admission. We designed directed acyclic graphs (DAGs) to guide a priori selection of potential confounding variables for the models, excluding mediating and collider variables^(15)^ (Figure 1S - Supplementary Material). We designed the DAGs using the online tool Dagitty (https://dagitty.net). We reported odds ratios (OR) and 95% confidence intervals (95% CI).

We aimed to assess the associations between IMV use and the onset of new morbidity at pediatric ICU discharge and 12-month mortality using an adjusted Cox regression model. However, the proportional hazards assumption, tested with Schoenfeld's test, was not satisfied. As a result, we used logistic regression models to assess these associations. To assess the association between IMV use and 12-month mortality, we adjusted the model for PRISM I score, pediatric ICU admission type, and FSS total score at admission. To assess the association of the development of new morbidity and 12-month mortality, we adjusted the model for PRISM I score, pediatric ICU admission type, FSS total score at admission, and IMV. Directed acyclic graphs were again used to guide variable selection (Figures 2S and 3S - Supplementary Material).

We planned to further explore possible causal mechanisms between IMV use, the onset of new morbidity, and 12-month mortality by performing a mediation analysis with IMV as the exposure of interest, new morbidity as the mediator, and 12-month mortality as the outcome. This would allow us to assess both the direct and indirect (i.e., mediated by new morbidity at pediatric ICU discharge) effects of IMV on 12-month mortality. However, because we did not find an association between IMV and 12-month mortality in the adjusted logistic regression model, we did not perform the mediation analysis.

In an exploratory analysis, we evaluated the association between IMV duration and the onset of new morbidity using multivariate logistic regression models for both definitions. Because ventilation duration may affect clinical outcomes nonlinearly, we used restricted cubic splines with four knots. We adjusted the model for PRISM I score, pediatric ICU admission type, and FSS total score at admission. We assessed goodness of fit and departure from linearity using Wald tests. We calculated ORs for time intervals based on visual inspection of the cubic splines. Finally, we evaluated the association between IMV duration and 12-month mortality using a multivariate logistic regression model, modeling IMV duration with restricted cubic splines (four knots) and adjusting for the same previous covariates.

A p value less than 0.05 indicates statistical significance for all analyses.

## RESULTS

During the study period, 729 patients were admitted to the pediatric ICU, of whom 423 were included. A total of 176 patients (41.6%) were mechanically ventilated during pediatric ICU stay (Figure 1).

**Figure 1:**
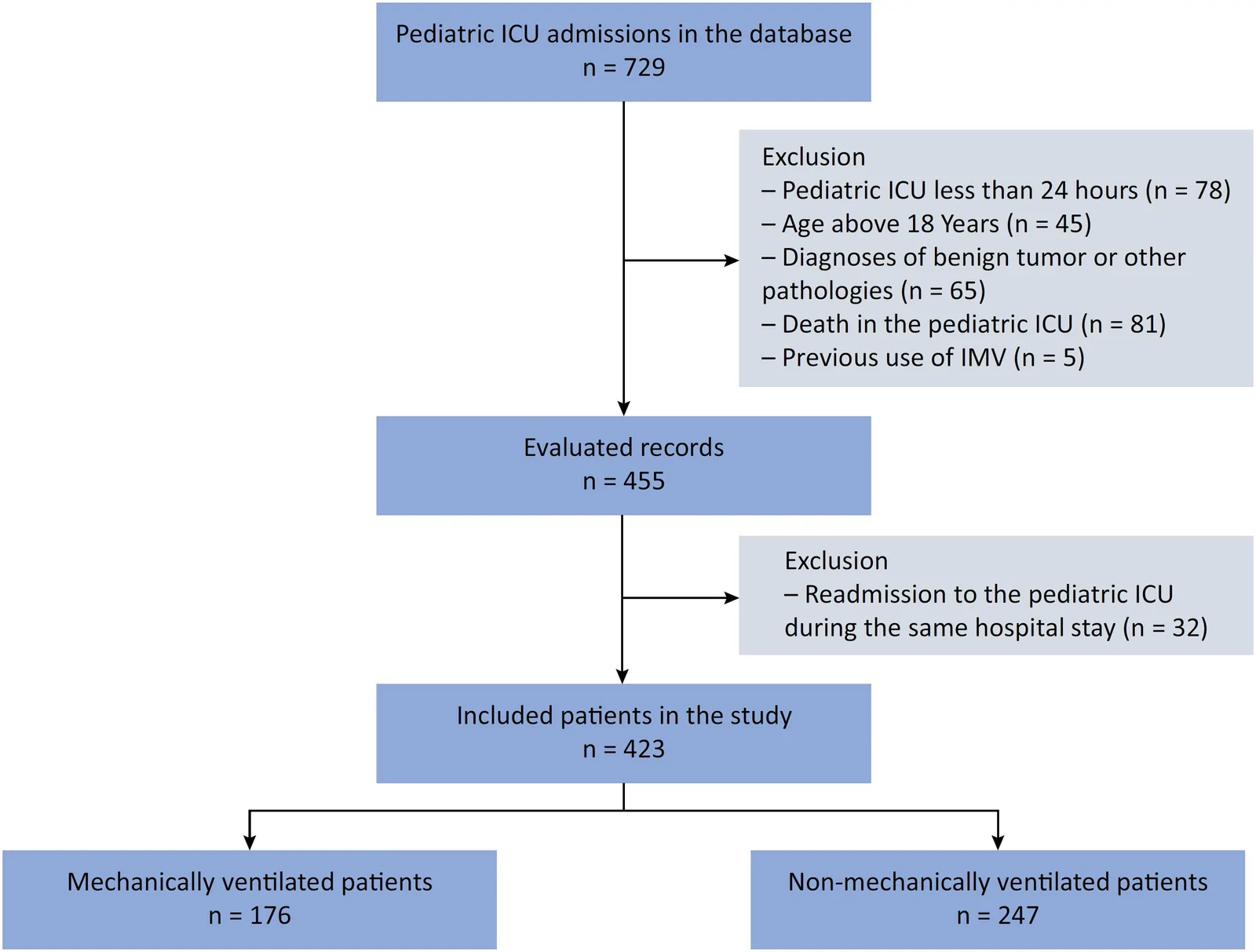
Flowchart showing patient inclusion.

ICU - intensive care unit; IMV - invasive mechanical ventilation.

Table 1 summarizes baseline characteristics. In both groups, most patients were diagnosed with solid tumors, particularly central nervous system tumors (Figure 2). Most patients had started cancer treatment before admission to the pediatric ICU, with chemotherapy being the most frequent modality in both groups (Table 1). During their pediatric ICU stay, 96 (54.5%) of IMV patients and 175 (70.8%) of non-IMV patients received anticancer treatment. Chemotherapy was the most frequent treatment among IMV patients (n = 48, [27.2%]), while surgery was the most frequent among non-IMV patients (n = 135, [54.6%]).

**Table 1:** Baseline characteristics of patients

		IMV (n = 176)	Non-IMV (n = 247)
Sex (male)		90 (51.1)	131 (53)
Age (years)		6.8 (3.2 - 12.1)	9.3 (3.4 - 13.9)
Type of cancer			
	Solid tumor	148 (84)	213 (86.2)
	Hematological disease	28 (16)	34 (13.7)
Metastasis (solid tumors)			
	No	61 (41,2)	91 (42,7)
	Yes	54 (36,4)	88 (31,3)
	No information	33 (22,2)	34 (15,9)
Previous treatment*			
	Chemotherapy	93 (52.8)	166 (67.2)
	Surgery	73 (41.4)	77 (31.1)
	Radiotherapy	35 (19.8)	35 (14.1)
	Target-therapy	4 (2.2)	7 (2.8)
	HSCT	4 (2.2)	3 (1.2)
	None	53 (30.1)	61 (24.6)
Admission type in the pediatric ICU			
	Surgical	57 (32.3)	151 (61.1)
	Medical	119 (67.6)	96 (38.8)
Source of admission			
	Wards	110 (62.5)	166 (67.2)
	Emergency department	46 (26.1)	37 (14.9)
	Outpatient clinic	10 (5.6)	35 (14.1)
	Hospital transfer	10 (5.6)	9 (3.6)
	PRISM I	1.9 (1 - 5)	0.7 (0.4 - 0.9)
	Pediatric ICU LOS (days)	17 (8 -31)	5 (3 - 7)
	Hospital LOS (days)	39.5 (23 - 63.7)	14 (8 - 28)
	Hospital outcome (death)	24 (13.6)	11 (4.4)

IMV - invasive mechanical ventilation; HSCT - hematopoietic stem cell transplantation; ICU - intensive care unit; PRISM I - Pediatric Risk of Mortality version I; LOS - length of stay.

* Some patients received more than one treatment modality. Results expressed as n (%) or median (interquartile range).

**Figure 2:**
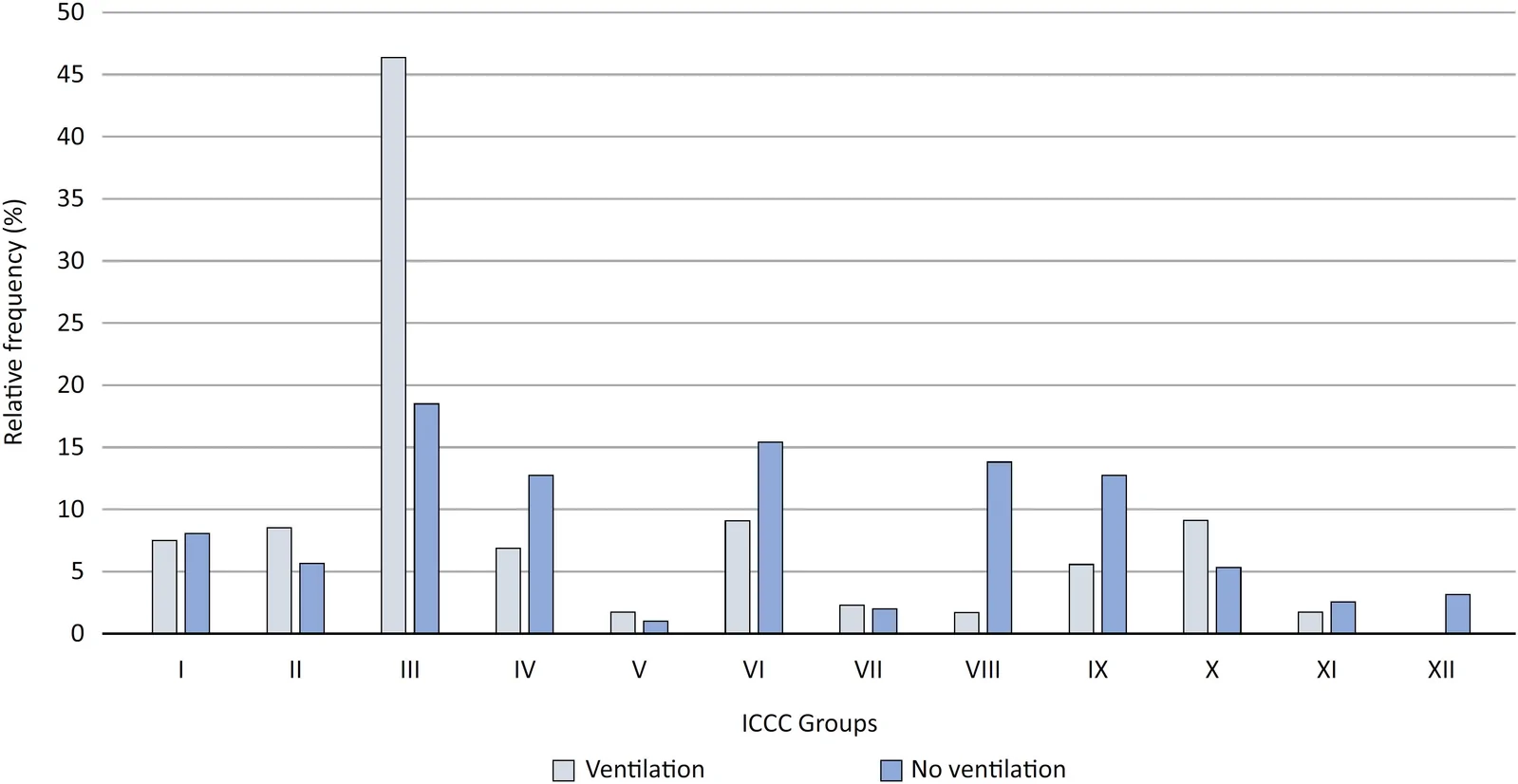
Relative frequencies of oncology diagnoses, based on the International Classification of Childhood Cancer for patients included in the study.

ICCC - International Classification of Childhood Cancer. Groups: I - leukemias; II - lymphomas; III - central nervous system tumors; IV - neuroblastoma; V - retinoblastoma; VI - renal tumors; VII - hepatic tumors; VIII - bone tumors; IX - soft tissue sarcomas; X - germ cell tumors; XI - other epithelial neoplasms; XII - unspecified.

The median IMV duration was 10 days (IQR 3 - 17). Of the 165 patients who received IMV via endotracheal tube, 83% were intubated at admission or within 24 hours afterward.

Medical admissions were more than double that of surgical admissions for mechanically ventilated patients, while surgical admissions were 1.5 time higher than medical admissions for non-mechanically ventilated patients. In both groups, most patients were transferred from the wards to the pediatric ICU (Table 1).

Regarding illness severity at admission, the PRISM I score was significantly higher in the mechanically ventilated patients. The median pediatric ICU LOS was three times longer for the mechanically ventilated patients, while the hospital LOS was twice as long (Table 1).

The Physiotherapy team evaluated all patients within 48 hours after admission and monitored them during their pediatric ICU stay, according to individual needs. Physiotherapy follow-up began a median of 3 days (IQR 2 - 4) after admission in both groups. However, ventilated patients had more sessions (18 days; IQR 7 - 35.7) than non-ventilated patients (2 days; IQR 1 - 4) (p < 0.01).

In both groups, most patients had normal functional capacity upon admission to the pediatric ICU, according to the FSS (Figure 3). However, the IMV group had a significantly higher total FSS score than the non-IMV group (Table 2 and Figure 4S [Supplementary Material]).

**Figure 3:**
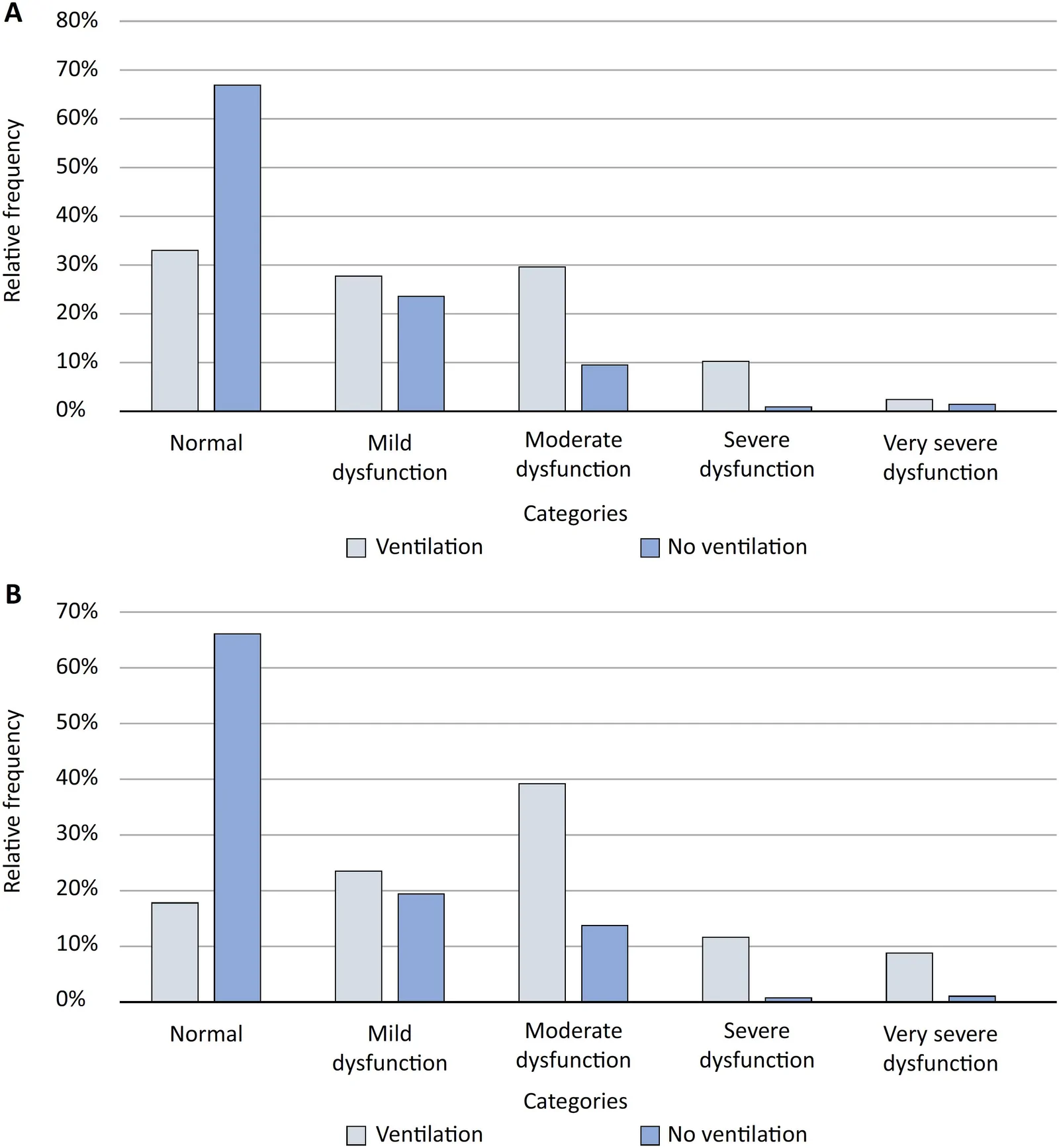
Distribution of patients according to the categories of the Functional Status Scale at admission (A) and at discharge (B) from the pediatric intensive care unit.

**Table 2:** Total score in the Functional Status Scale at admission and at discharge from the pediatric intensive care unit, affected domains of Functional Status Scale at discharge, and frequencies of new morbidities

		IMV (n = 176)	Non-IMV (n = 247)	p value
FSS at admission		9 (7 - 11)	7 (6 - 8)	< 0.01*
FSS at discharge		11 (8 - 14)	7 (6 - 8)	< 0.01*
Affected domains at discharge				
	Feeding	61 (34.6)	17 (6.8)	< 0.01†
	Motor functioning	54 (30.6)	6 (2.4)	< 0.01†
	Respiratory status	29 (16.4)	1 (0.4)	< 0.01†
	Mental status	15 (8.5)	0	< 0.01†
	Communication	14 (7.9)	0	< 0.01†
	Sensory functioning	12 (6.8)	0	< 0.01†
New morbidity, definition 1		69 (39.2)	12 (4.8)	< 0.01†
New morbidity, definition 2		95 (53.9)	20 (8.1)	< 0.01†

IMV - invasive mechanical ventilation; FSS - Functional Status Scale. New morbidity, definition 1: an increase of 3 or more points in the total score of Functional Status Scale between admission and discharge from the pediatric intensive care unit. New morbidity, definition 2: an increase of 2 or more points in any domain of the Functional Status Scale between admission and discharge from the pediatric intensive care unit.

* Mann-Whitney U test

† chi-squared test. Results expressed as median (interquartile range) or n (%).

At discharge from the pediatric ICU, the total FSS score remained significantly higher in the IMV group (Table 2 and Figure 4S [Supplementary Material]). There was nearly an 8-fold increase in the frequency of patients classified as having very severe dysfunction in the IMV group, rising from 1.7% at admission to 8.5% at pediatric ICU discharge (Figure 3).

The frequency of new morbidities was higher in patients who received mechanical ventilation, according to both definitions (IMV: n = 69, [39.2%] *versus* no IMV: n = 12, [4.8%] on definition 1; IMV: n = 95, [53.9%] *versus* No IMV: n = 20, [8.1%] on definition 2) (Table 2). The main affected domains were feeding and motor functioning in both mechanically ventilated and non-ventilated patients (Table 2).

IMV use was associated with the development of new morbidity, according to definition 1, in both unadjusted (OR 12.6; 95%CI 6.8 - 25.4; p < 0.001) and adjusted models (OR 13.0; 95%CI, 6.6 - 27.3; p < 0.001). Invasive mechanical ventilation use was also associated with new morbidity according to definition 2 in both models (unadjusted OR 13.3; 95%CI 7.9 - 23.5; p < 0.001; adjusted OR 10.3; 95%CI 5.8 - 19.0; p < 0.001).

The onset of new morbidities during pediatric ICU stay was associated with higher 12-month mortality after discharge (Figures 4A and 4B). For both definitions, new morbidities were associated with increased 12-month mortality both in unadjusted (definition 1, OR 2.6; 95%CI 1.6 - 4.3; p < 0.01; definition 2, OR 2.9; CI95% 1.8 - 4.5; p < 0.01) and adjusted models (definition 1, OR 2.1; 95%CI 1.2 - 3.7; p = 0.013; definition 2, OR 2.1; 95%CI 1.2 - 3.6; p < 0.001). Invasive mechanical ventilation use was associated with increased 12-month mortality (Figure 4C) only in the unadjusted model (unadjusted OR 2.0; 95%CI 1.3 - 3.1; p < 0.01; adjusted OR 1.4; 95%CI, 0.9 - 2.3; p = 0.15).

**Figure 4:**
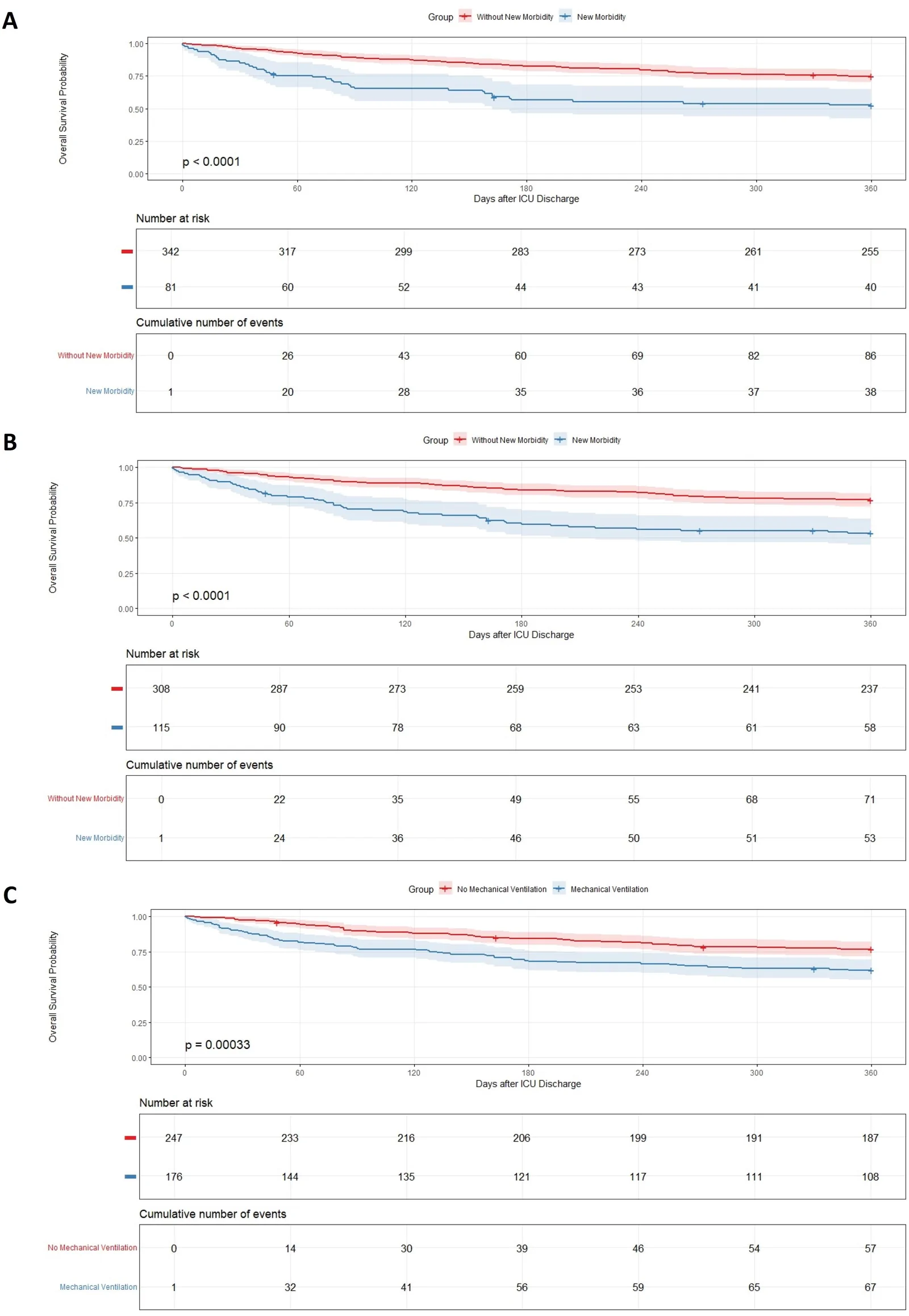
Mortality of patients based on the onset of new morbidity at pediatric intensive care unit discharge and on invasive mechanical ventilation use.

(A) New morbidities are defined as an increase of three or more points in the total score of Functional Status Scale between admission and discharge from the pediatric intensive care unit. (B) New morbidities are defined as an increase of two or more points in any domain of the Functional Status Scale between admission and discharge from the pediatric intensive care unit.

Invasive mechanical ventilation duration was nonlinearly associated with the onset of new morbidity for both definitions (p < 0.01 for both). For definition 1, visual inspection of the restricted cubic splines showed an accelerated increase in odds during the first 10 days of IMV (Figure 5S - Supplementary Material). Specifically, the odds of developing a new morbidity were nearly 11-fold higher at 10 days compared to 1 day of IMV (adjusted OR = 10.25; 95%CI 2.40 - 43.78). For definition 2, visual inspection of the restricted cubic splines showed a sharp increase in odds during the first 7 to 10 days of respiratory support, followed by a more gradual upward trend for longer durations (Figure 5S - Supplementary Material). Specifically, the odds of developing a new morbidity according to definition B were nearly 11-fold higher at 7 days compared to 1 day of IMV (adjusted OR = 10.95; 95%CI 2.90 - 41.40). Beyond the first week, the odds continued to rise, with 14 days of ventilation more than doubling the odds compared to 7 days (adjusted OR = 2.71; 95%CI 1.46 - 5.01). On the other hand, there was no association between IMV duration and 12-month mortality (p = 0.12) (Figure 5S - Supplementary Material).

## DISCUSSION

In this study, we identified a significant association between IMV use during pediatric ICU stay and lower functional capacity at pediatric ICU discharge in pediatric oncology patients. New morbidity was more frequent in mechanically ventilated patients under both definitions used. Additionally, new morbidity was associated with higher 12-month post-discharge mortality, whereas IMV itself was not.

Previous studies have reported functional decline following critical illness in children, with prevalence rates ranging from 3.5% to 68%, depending on patient characteristics and illness severity.^(6;16-18)^ In pediatric patients without respiratory diagnoses, one study showed a prevalence of new morbidity of 77.2%, and an association between muscle atrophy and the development of new morbidity at pediatric ICU discharge.^(19)^ In our cohort, 19.1% of patients exhibited decreased functional capacity at pediatric ICU discharge, consistent with previous findings.^(20)^ Ventilated patients had an eightfold higher frequency than non-ventilated patients, suggesting the impact of IMV on functional capacity.

Among critically ill children in general, IMV is associated with functional decline, particularly with longer IMV durations.^(6,21,22)^ In a previous study of critically ill children with cancer, IMV at pediatric ICU admission increased the odds of new morbidity at hospital discharge by 2.9 times.^(23)^ In our cohort, the odds of new morbidities were 13 times higher in IMV patients after adjustment for confounders, highlighting the increased risk of functional impairment after pediatric ICU discharge in mechanically ventilated oncology patients. The odds of new morbidity were 11 times higher at 7 and 10 days of IMV compared to the first day and doubled at 14 days compared to 7 days. The cumulative burden of immunosuppression, infection risk, organ dysfunction, and ventilator-related complications likely accounts for these elevated odds.^(2,24,25)^

Invasive mechanical ventilation is reported as a predictor of both short- and long-term mortality in critically ill children,^(26)^ especially in oncology patients^(7,27)^ and those who develop new morbidity.^(23)^ In our cohort, 13.6% of mechanically ventilated patients died in the hospital after pediatric ICU discharge. The association between IMV and 12-month mortality was not significant after adjustment for confounders, likely because unmeasured clinical factors in the post-discharge period, including disease progression, oncological treatment changes or interruptions, and unplanned hospitalizations, dominate late mortality risk in this population.^(26,28)^ The direct effect of IMV on late mortality, after accounting for the mediation through new morbidity, may be small or absent in pediatric ICU survivors.

Feeding and motor domains are the most affected functional areas after pediatric critical illness.^(18,21,22)^ Likewise, our study found greater impairments in these domains among children with cancer, supporting the findings of previous studies.^(20,23)^ These results highlight the importance of early multidisciplinary intervention, particularly in motor rehabilitation and swallowing therapy, to facilitate functional recovery and improve long-term outcomes. In our study, both mechanically ventilated and non-ventilated patients initiated physiotherapy follow-up early (median 3 days after admission), and all received care during their pediatric ICU stay, based on clinical conditions and specific rehabilitation needs. Mechanically ventilated patients received more sessions. However, we did not analyze the effects of session frequency or procedures on functional outcomes because interventions varied widely and no standardized institutional protocol existed. The wide variability in patients’ age ranges and functional demands, arising from different developmental stages and limitations caused by various oncological diseases, made it difficult to standardize physiotherapy care and, consequently, to establish associations with functional outcomes.

This study has some limitations. First, its retrospective, single-center design limits generalizability, and data from a tertiary cancer center may not reflect lower-complexity settings. Second, we excluded patients who died in the pediatric ICU, which may have introduced selection bias by excluding patients with greater severity. However, the primary outcome of interest was the onset of new morbidity at pediatric ICU discharge. It is possible that the association between IMV and the composite outcome of mortality and functional decline would be even greater than the association with functional decline alone in survivors. However, this analysis was outside the scope of this study. Third, we did not collect information on cancer progression or changes in oncologic treatment after pediatric ICU discharge, which could have affected the 12-month mortality outcomes. Fourth, IMV indications were not systematically recorded, precluding analysis of whether functional impairment in feeding and motor domains reflected IMV effects or underlying disease, particularly central nervous system tumor involvement. Fifth, although the FSS is widely used in general pediatric critical care studies, its sensitivity for detecting all functional dimensions in hospitalized children with cancer remains uncertain. To minimize bias, we applied two previously published FSS-based morbidity definitions, with a single researcher responsible for classification.

## CONCLUSION

Critically ill children with cancer who received invasive mechanical ventilation during their pediatric intensive care unit stay had a 13-fold increased odds of new morbidities at discharge, with a non-linear association between invasive mechanical ventilation duration and morbidity onset. The development of new morbidities doubled the odds of death within 12 months after pediatric intensive care unit discharge. Future prospective studies should include systematic post-discharge follow-up and evaluate the impact of multidisciplinary rehabilitation on long-term functional recovery and mortality in this population.

## Data Availability

Data is available on demand from referees.
