# Supplementary material for: Invasive mechanical ventilation, functional decline at pediatric intensive care unit discharge, and post-discharge mortality in critically ill pediatric cancer patients: a retrospective cohort study
Source: Crit Care Sci. 2026 Sep 17;38:e20260121. doi: 10.62675/2965-2774.20260121 (PMC13690030; doi:10.62675/2965-2774.20260121)
Supplement: Supplementary file 1 [file 2965-2774-ccsci-38-e20260121-suppl01.pdf]

Critical Care Science
https://doi.org/10.62675/2965-2774.20260121

SUPPLEMENTARY MATERIAL

Invasive mechanical ventilation, functional decline at pediatric intensive care unit discharge, and post-discharge mortality in critically ill pediatric cancer patients: a retrospective cohort study

Beatriz Silva Menezes da Cunha-Martins[1], Rachel Silva Menezes da Cunha[1], Vanessa Soares Lanziotti[2], Antonio Paulo Nassar Jr[3]

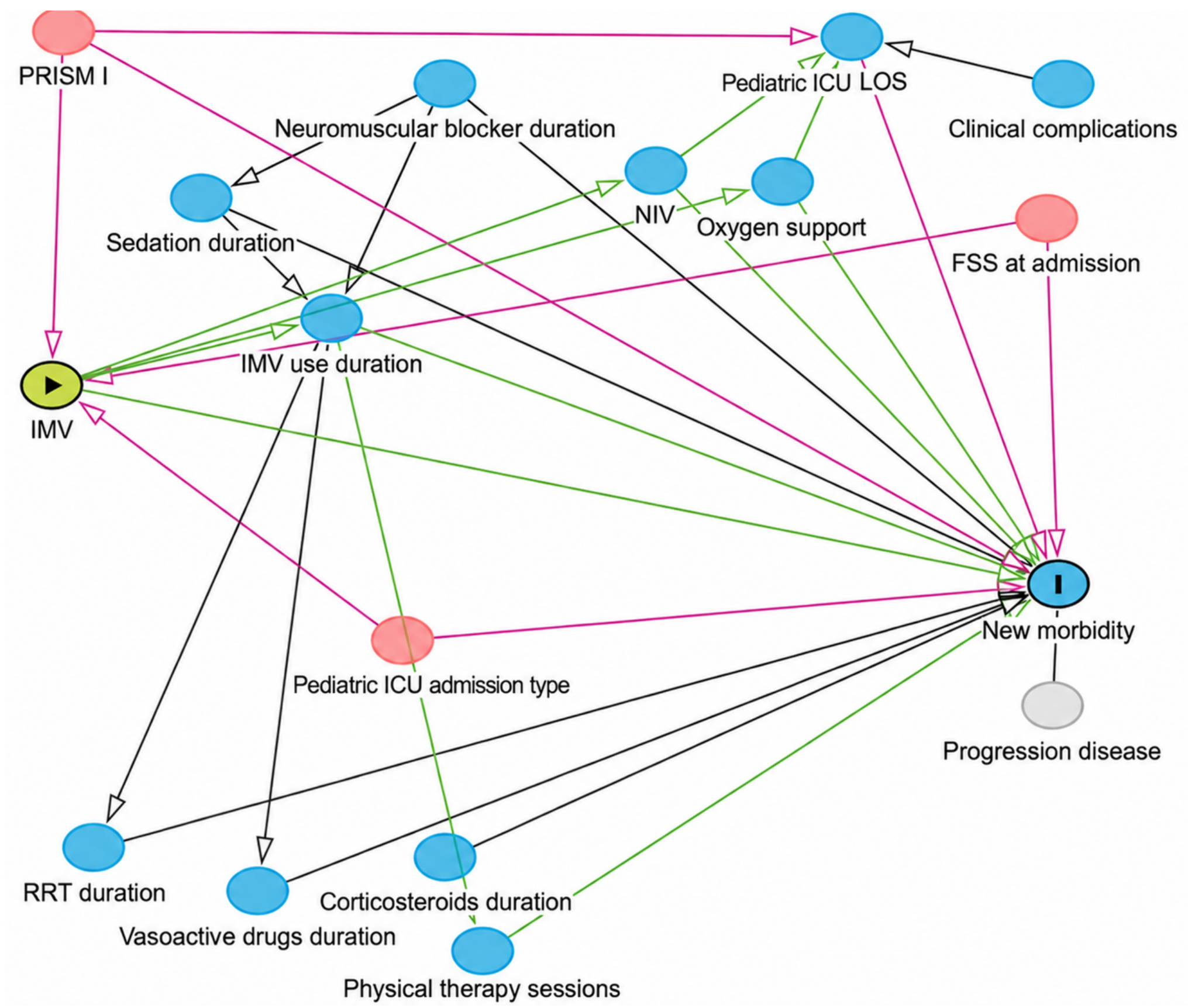

PRISM I - Pediatric Risk of Mortality version I; ICU - intensive care unit; LOS - length of stay; NIV - noninvasive ventilation; FSS - Functional Status Scale; IMV - invasive mechanical ventilation; RRT - renal replacement therapy.

Figure 1S - Directed acyclic graph guiding the selection of variables included in the multiple logistic regression to assess the association between invasive mechanical ventilation and the onset of new morbidity in critically ill children admitted to the pediatric intensive care unit.

This is an open access article under the CC BY license (https://creativecommons.org/licenses/by/4.0/).

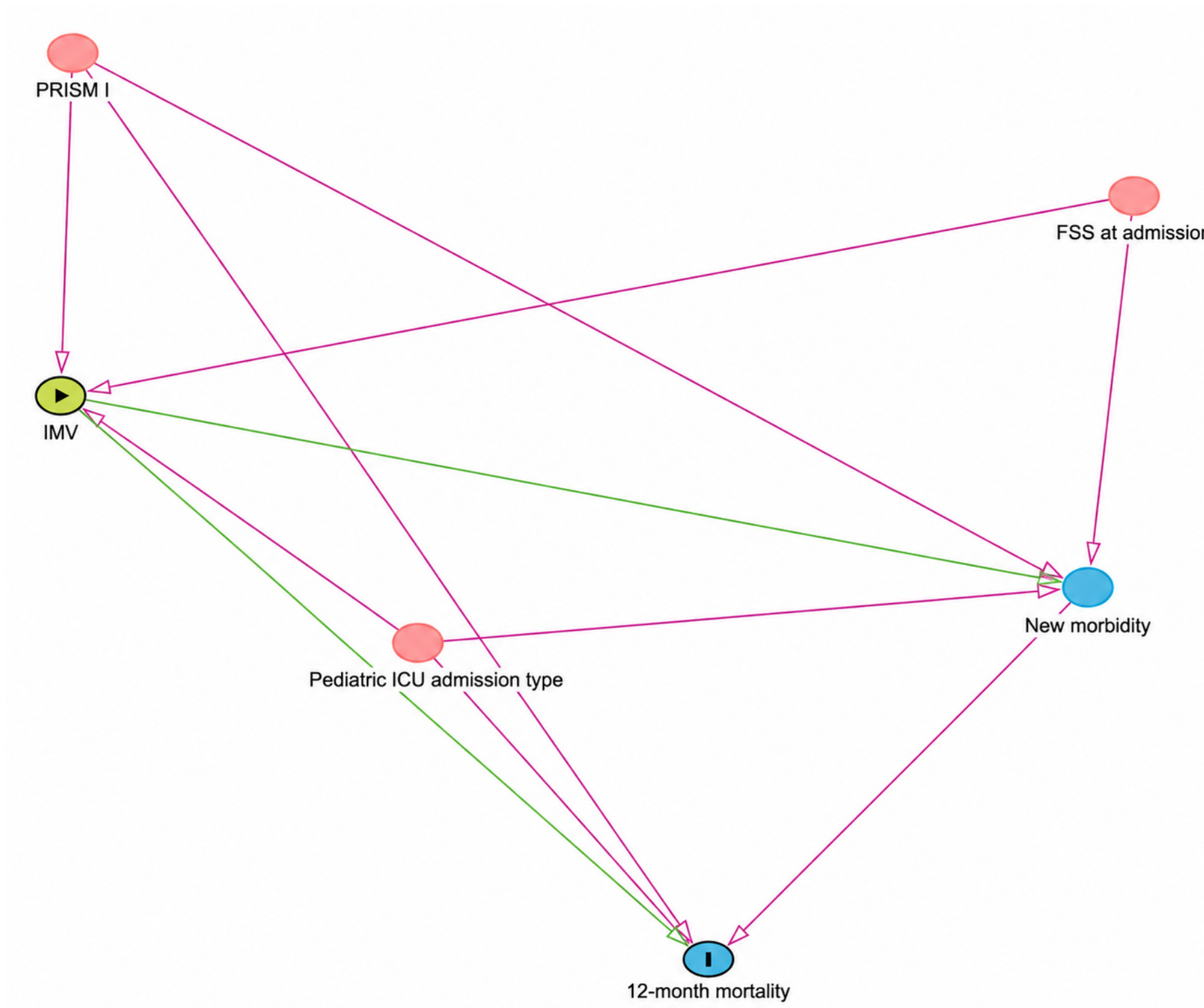

PRISM I - Pediatric Risk of Mortality version I; FSS - Functional Status Scale; IMV - invasive mechanical ventilation; ICU - intensive care unit.

Figure 2S - Directed acyclic graph guiding the selection of variables included in the multiple logistic regression to assess the association between invasive mechanical ventilation and 12-month mortality in critically ill children admitted to the pediatric intensive care unit.

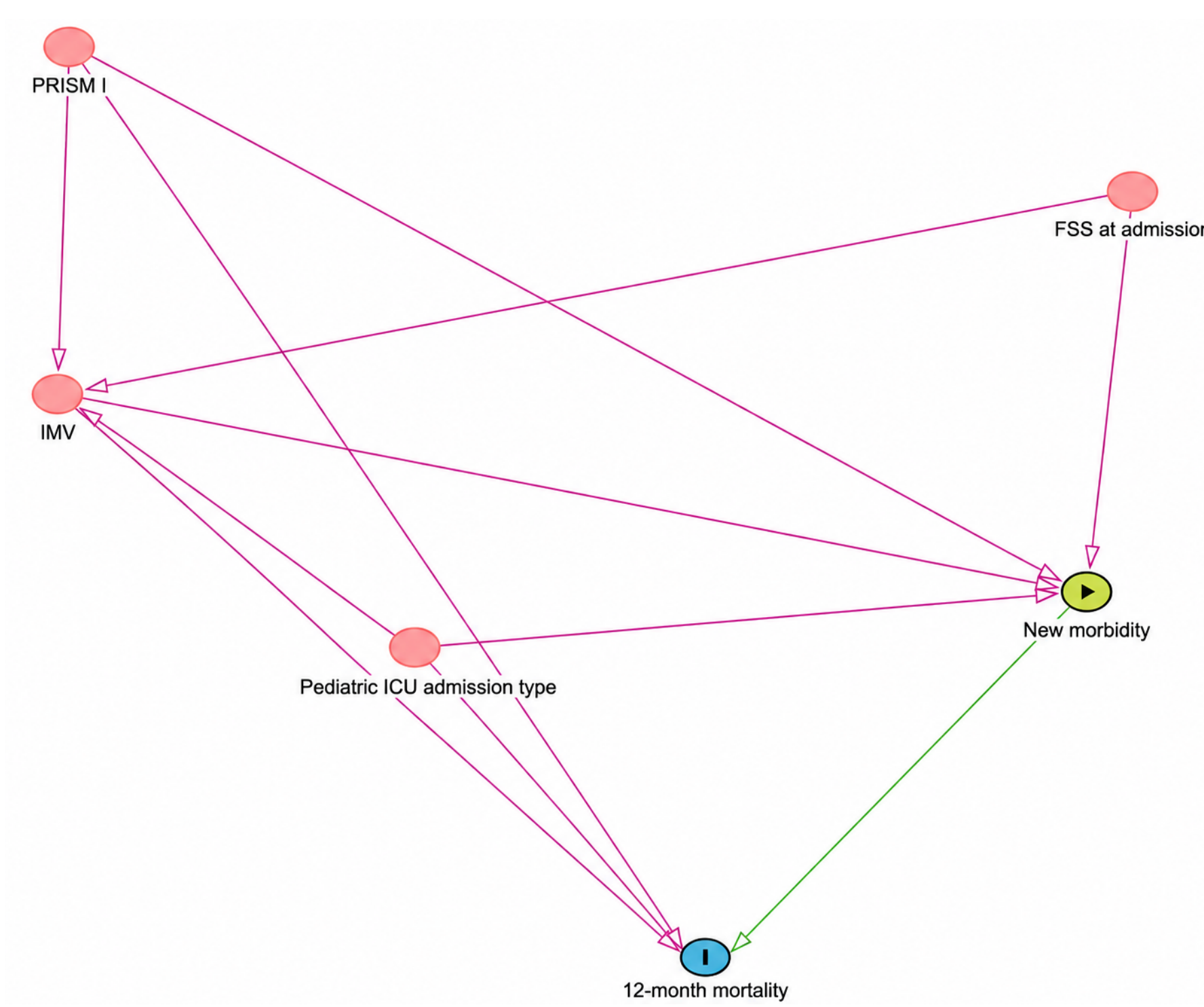

PRISM I - Pediatric Risk of Mortality version I; FSS - Functional Status Scale; IMV - invasive mechanical ventilation; ICU - intensive care unit.

Figure 3S - Directed acyclic graph guiding the selection of variables included in the multiple logistic regression to assess the association between the onset of new morbidity and 12-month mortality in critically ill children admitted to the pediatric intensive care unit.

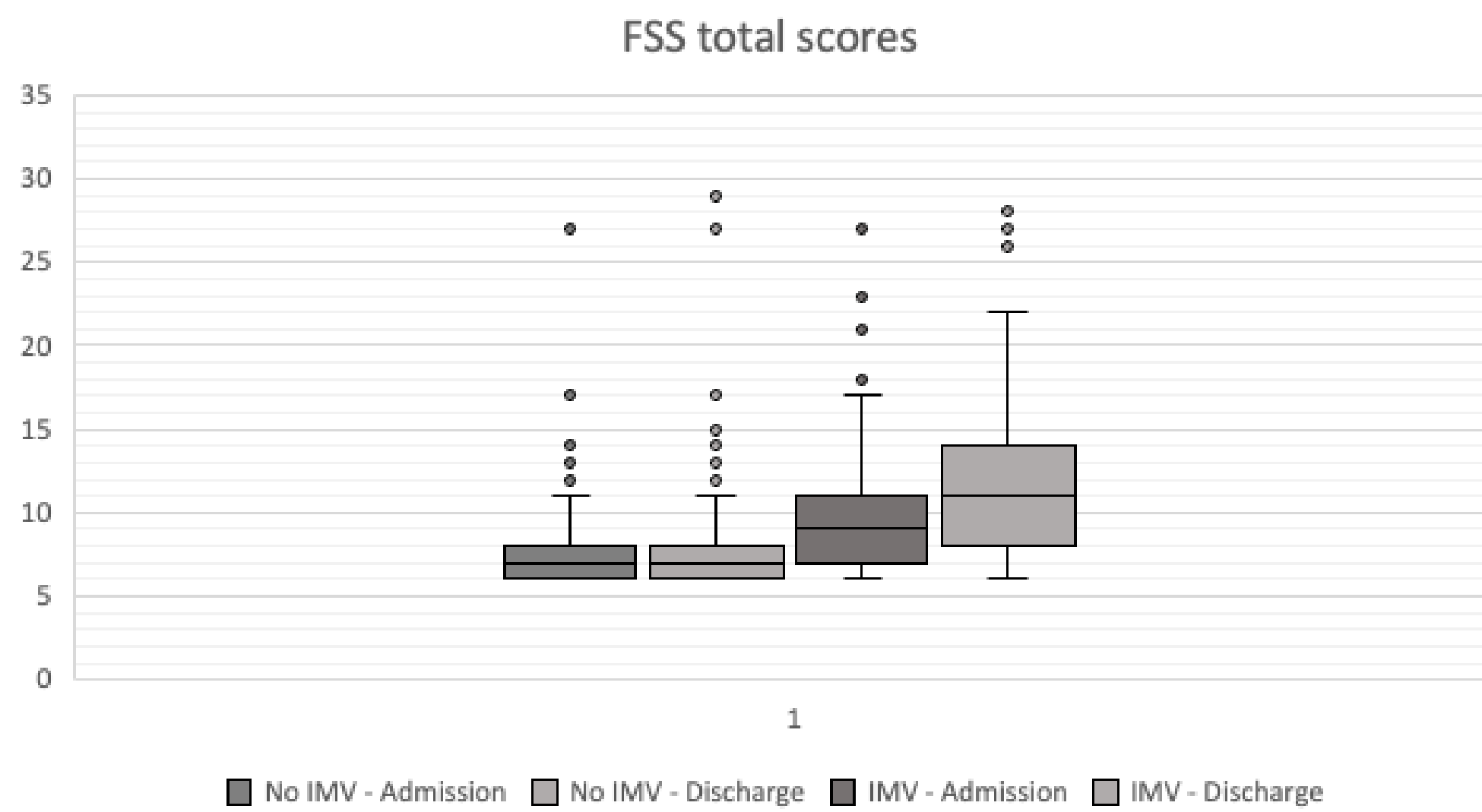

FSS - Functional Status Scale; IMV - invasive mechanical ventilation.

Figure 4S - Boxplot of total Functional Status Scale scores in ventilated and non-ventilated patients at admission and at discharge from the pediatric intensive care unit.

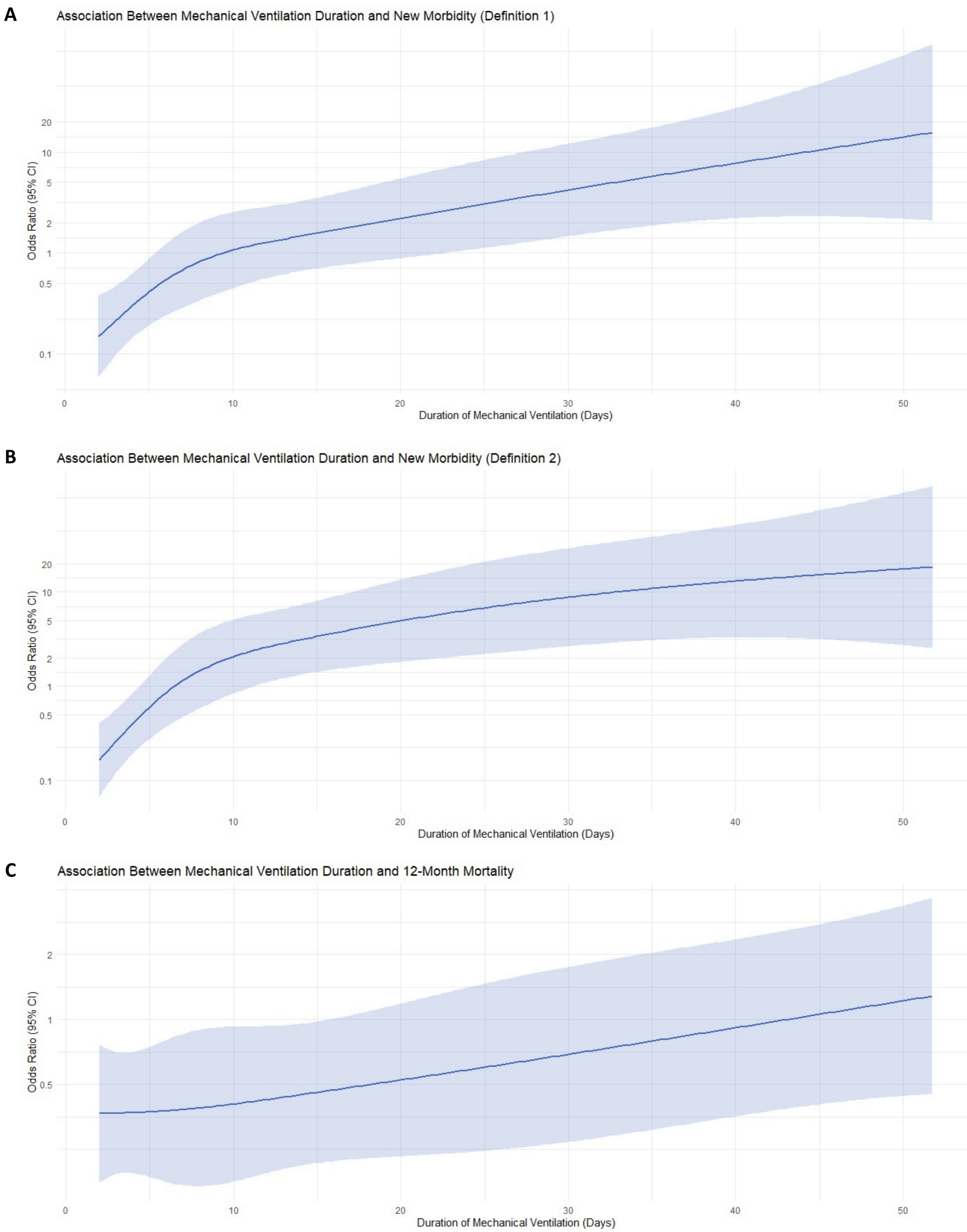

95%CI - 95% confidence interval.

Figure 5S - Non-linear association of duration of invasive mechanical ventilation and the onset of a new morbidity, and duration of invasive mechanical ventilation and 12-month mortality.

(A) New morbidities are defined as an increase of 3 or more points in the total score of the Functional Status Scale between admission and discharge from the pediatric intensive care unit. (B) New morbidities are defined as an increase of 2 or more points at any domain of Functional Status Scale between admission and discharge from the pediatric intensive care unit.
